# Auditory sustained field responses to periodic noise

**DOI:** 10.1186/1471-2202-13-7

**Published:** 2012-01-06

**Authors:** Sumru Keceli, Koji Inui, Hidehiko Okamoto, Naofumi Otsuru, Ryusuke Kakigi

**Affiliations:** 1 Department of Integrative Physiology, National Institute for Physiological Sciences, Nishigohnaka 38, Myodaiji, Okazaki City, 444-8585, Japan; 2 Department of Physiological Sciences, School of Life Sciences, The Graduate University for Advanced Studies, Hayama, Kanagawa, Japan

## Abstract

**Background:**

Auditory sustained responses have been recently suggested to reflect neural processing of speech sounds in the auditory cortex. As periodic fluctuations below the pitch range are important for speech perception, it is necessary to investigate how low frequency periodic sounds are processed in the human auditory cortex. Auditory sustained responses have been shown to be sensitive to temporal regularity but the relationship between the amplitudes of auditory evoked sustained responses and the repetitive rates of auditory inputs remains elusive. As the temporal and spectral features of sounds enhance different components of sustained responses, previous studies with click trains and vowel stimuli presented diverging results. In order to investigate the effect of repetition rate on cortical responses, we analyzed the auditory sustained fields evoked by periodic and aperiodic noises using magnetoencephalography.

**Results:**

Sustained fields were elicited by white noise and repeating frozen noise stimuli with repetition rates of 5-, 10-, 50-, 200- and 500 Hz. The sustained field amplitudes were significantly larger for all the periodic stimuli than for white noise. Although the sustained field amplitudes showed a rising and falling pattern within the repetition rate range, the response amplitudes to 5 Hz repetition rate were significantly larger than to 500 Hz.

**Conclusions:**

The enhanced sustained field responses to periodic noises show that cortical sensitivity to periodic sounds is maintained for a wide range of repetition rates. Persistence of periodicity sensitivity below the pitch range suggests that in addition to processing the fundamental frequency of voice, sustained field generators can also resolve low frequency temporal modulations in speech envelope.

## Background

Auditory stimulation with a brief sound elicits several transient evoked responses that are time-locked to the onset of the stimulus. When the sound stimulus is long enough, the transient responses are followed by the stimulus locked DC-shift sustained response, which persists for the duration of the sound [1,2] and returns to baseline shortly after the stimulus offset [2,3]. Sustained response is named as sustained potential (SP) when recorded by electroencephalography and as sustained field (SF) when recorded by magnetoencephalography (MEG). Picton et al. [4,5] investigated the human SP in detail and showed that compared to the onset responses, tone-evoked SP amplitudes reflect stimulus intensity variations more accurately and are relatively insensitive to increasing stimulus presentation rates. Based on these findings, Picton et al. [5] suggested that sustained potentials are more closely related to sensory analysis of the auditory input than the transient responses. Recent MEG recordings under masking paradigms revealed that SF responses are also strongly influenced by top-down attentional neural activity [6] and might reflect subjects' awareness of the test sounds [7].

Sustained responses can be elicited by various sound stimuli other than tones such as click trains [8], speech sounds [9], and noises [10] and the response amplitudes vary with the stimuli. In fact, an important aspect of sustained responses has been revealed by the analysis of the SF amplitudes evoked by click trains. Using MEG, a non-invasive technique that combines high temporal acuity with good spatial resolution, Gutschalk et al. [8] showed that regular interval click trains evoke significantly larger SF responses than do their irregular counterparts. Based on this finding, they described two anatomically and functionally distinct components for SF; one highly sensitive to temporal regularity and the other sensitive mainly to sound level [8]. Vowel stimulation was also reported to evoke enhanced sustained responses compared to intensity-matched pure tones [9] and acoustically-matched noise [10]. Dipole analysis of the evoked potential waveforms revealed a vowel-specific SP component in addition to a common component, which responds to both noise and vowels [10]. In an attempt to clarify the relationship between the regularity-sensitive and vowel-sensitive components of the sustained responses, Gutschalk and Uppenkamp [11] investigated the SF/SP responses to periodicity and vowel quality contrasts. The contrasts were introduced by modification of the formant structure (vowel vs. non-vowel) and/or the repetition regularity (periodic vs. jittered) of a set of damped sinusoids that produce easily identifiable vowels A, E, I, O, or U with a repetition rate of 83.3 Hz. The sustained responses evoked by these stimuli showed a clear functional separation of the regularity- and vowel-specific components, i.e. former responding mainly to periodic stimuli (vowel or non-vowel) and latter to vowels (periodic or jittered). Co-localization of the source generators of these functionally distinct components in the auditory cortex led the authors suggest that sustained responses reflect a stage of speech processing where the fundamental frequency (F0) and formant structures are detected [11]. F0 of speech signals reflects the lowest periodic component of vocal fold vibrations and perceived as voice pitch. Speech also contains periodic or quasiperiodic fluctuations below the F0 of voice, which carry information on manner of articulation, voicing, vowel identity and prosody [12]. As these low frequency temporal modulations are crucial for speech intelligibility [13,14] (for a review see [15]), the role of periodicity-sensitive SF generators in speech processing should not be limited to detection of the voice pitch.

Previous studies on sustained responses to periodic stimuli present diverging results regarding the lower limit of periodicity detection. Periodic click train evoked SF responses amplitudes were shown to decrease significantly as the inter-click intervals of the periodic click trains are increased from 5 ms (200 Hz) to 100 ms (10 Hz) [8] and this decreasing trend was suggested to follow the cessation of the pitch perception that is produced by repetition rates greater than 30 Hz [8]. The amplitudes of periodic vowel-evoked SF, on the other hand, do not change across the repetition frequencies of 9 to 113 Hz [16]. The discrepancy between the results appears to be based on stimulus related factors. In case of click train stimulation, the decay of SF amplitudes might be due to the sound energy differences among the different F0 click trains. As the stimulus trains consist of constant intensity clicks that are repeated at a period of 1/F0, the sound energy would decrease at low repetition rates. In case of vowel stimulation, the stimuli were matched with respect to root mean square amplitudes because the temporal envelope of vowel stimuli resembles that of click trains as the vowels were built on a glottal pulse waveform [17] which permitted modification of the repetition frequency by changing the length of closed phase durations. The steady SF amplitudes in response to energy matched F0 conditions however, may be limited to the vowel stimuli. As vowel-evoked SF responses contain a distinct component in addition to the periodicity sensitive component [11], the use natural sounding vowels might have disguised the true nature sustained responses to low repetition rates. In order to clarify whether the periodicity detection persists below the pitch range, it is necessary to reproduce the low frequency fluctuations in an energy-controlled manner but without speech related spectral clues.

Repeating frozen noise (RFN) consists of repetition of the same noise segment (frozen noise) without pause and the reciprocal of the segment length defines the repetition rate. First used to explore the lower limits of auditory frequency analysis [18], RFN provides a model stimulus for studying perception of periodic sounds [19]. As the repetitive element is noise and there are no silent periods between the consecutive segments, it is possible to prepare equal energy stimuli for a wide range of repetition rates. In addition to ease of intensity control, their flat harmonic spectra make RFNs especially suitable to investigate the lower limits of periodicity detection and their flat amplitude envelope leads to stable SF responses. RFNs have been utilized in a number of psychophysical and electrophysiological studies investigating perception of faint events and echoic memory [20-23]. Perceptual characteristics of RFN has been described in three categories: infrapitch group (< 20 Hz) is perceived as "whooshing" (< 4 Hz) or "motorboating" (4 - 20 Hz), pure pitch group (> 100 Hz) produces a clear pitch sensation, and intermediate group (20 - 100 Hz) is perceived as noisy pitch [18,19,24]. In this study, we used RFN stimuli from infrapitch (5 and 10 Hz), pitch (200 and 500 Hz), and intermediate groups (50 Hz) with white noise as control (For sample audio files, see Additional file 1). We chose the 5-, 50-, and 500 Hz RFNs in order to investigate the effect of periodicity in a wide range while we used the 10- and 200 Hz conditions to compare our results with the previous report by Gutschalk et al. [8] where SF response amplitudes were shown to decrease at low repetition rates.

In this study, in order to investigate the effect of repetition rate without confounds of stimulus-specific features and energy differences, we investigated the auditory sustained fields elicited by white noises and RFNs that had different repetition rates (5, 10, 50, 200, and 500 Hz).

## Methods

### Subjects

The experiment was performed on twelve (three females) healthy, right-handed volunteers, aged between 27 and 48 years (36 ± 6). The study was approved in advance by the Ethics Committee of the National Institute for Physiological Sciences of Japan and written consent was obtained from all the subjects.

### Stimulus and recordings

**Figure 1:**
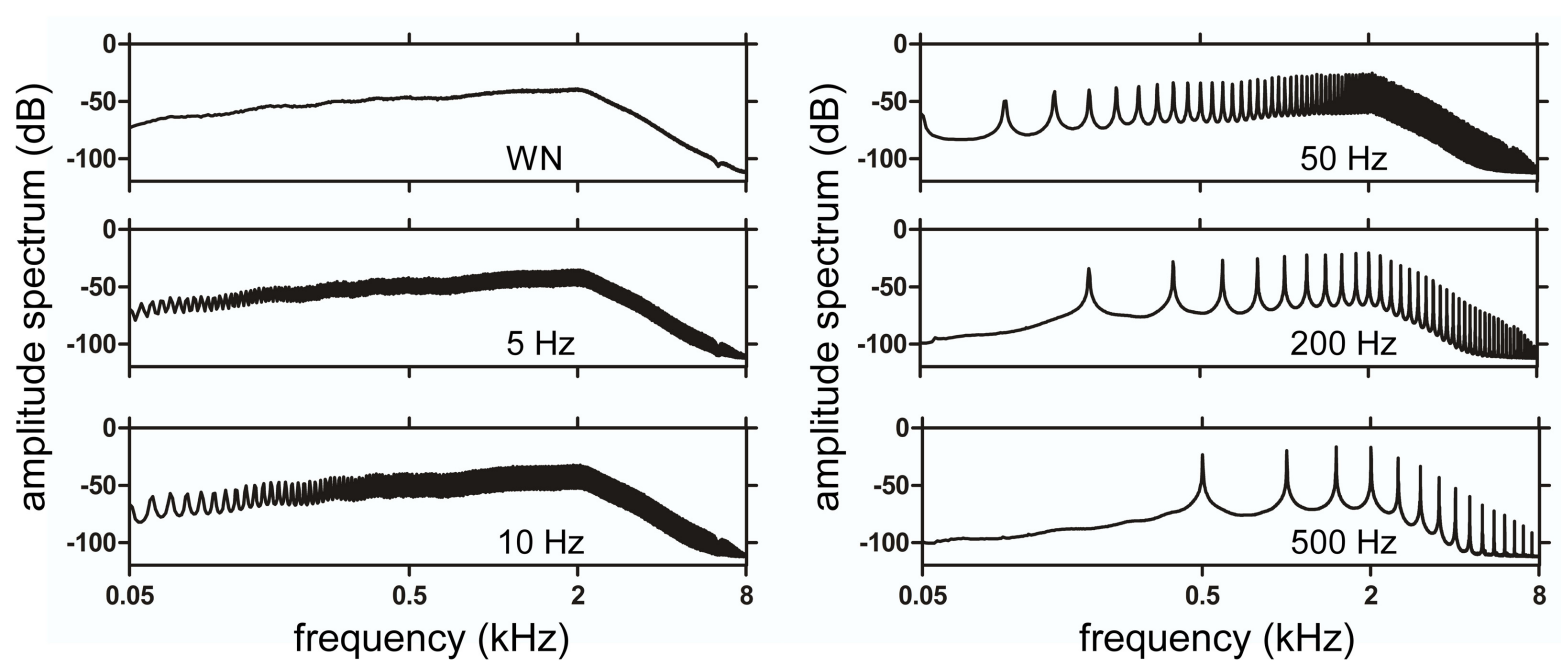
**Stimuli**. Amplitude spectra of the white noise (WN), 5-, 10-, 50-, 200-, and 500-Hz repeating frozen noises (RFNs). Because of our sound delivery system, all the sound spectra exhibit low-pass filtered characteristics. For sample audio files, see Additional file 1.

A long sequence of random numbers of between -1 and 1 was created at a 32 kHz sampling rate, and segments to be recycled were selected randomly from non-overlapping regions of the main sequence. For each rate condition, a segment of appropriate length (i.e. 20 ms for 50 Hz) was repeated without pause to make a one-second long stimulus. A set of two hundred stimuli was prepared for white noise (WN) and each rate condition (5, 10, 50, 200, and 500 Hz). The total number of stimuli used in the experiments was 1200. The stimuli in the WN set did not contain any repeating segments and served as control stimuli. To test the performance of our sound delivery system, which consists of insert earphones (E-A-RTONE 3A, Aearo Company Auditory Systems, Indianapolis, IN) connected to foam plugs (E-A-RLINK, Aearo Company Auditory Systems, Indianapolis, IN) by plastic sound tubes, we recorded all the stimuli at the earpiece. The recorded waveforms retained the repeating pattern of random fluctuations. The spectra that were computed over all the stimuli of each set showed a harmonic comb structure with high amplitude partials corresponding to integer multiplies of the fundamental frequency (Figure 1). The decrease of the spectral envelope towards high frequencies reflects the frequency response characteristics of the sound delivery system. A-weighted equivalent continuous sound level was measured over each second, while all the stimuli were played without pause, using a sound level meter (NL-22, Rion Co. Ltd., Tokyo, Japan) at the earpiece. The analysis of sound level values (dBA) confirmed that the stimuli had equal energy. The mean and standard deviation of sound levels with reference to the control WN was 0.1 ± 0.17, 0.1 ± 0.3, 0.04 ± 1, -0.1 ± 1, and 0.01 ± 1.5 dBA for 5, 10, 50, 200, and 500 Hz, respectively. During the experiments, the stimuli were delivered in a random order at 35 dB sensation level. A randomly selected stimulus from the 200 Hz set was used for threshold measurements. Each stimulus was presented only once with an interstimulus interval of 1500 ms.

Auditory evoked fields (AEFs) were recorded with a helmet-shaped 306 channel MEG system (Vector-view, ELEKTA, Neuromag, Helsinki, Finland) with 102 identical triple sensor elements. We analyzed MEG signals recorded by 204 planar-type gradiometers, which detect the signals directly beneath the sensors. The recordings were done in a magnetically shielded room while subjects were watching a subtitled silent movie of their own choice. The signals were recorded with a band-pass filter of 0.03-200 Hz and digitized at 1001 Hz. Electrooculograms were recorded to monitor the ocular artifacts. Evoked fields were averaged offline and epochs containing amplitude values of more than 2700 fT/cm or amplitude changes of more than 800 fT per sample were discarded. After the rejection, more than 187 trials were averaged for each condition. Prior to trigger based offline averaging for each subject, the continuous magnetic field and electrooculogram data were viewed simultaneously in order to find the magnetic field epochs that coincide with the blink artifacts on the electrooculograms. The identified magnetic field epochs were averaged separately in order to estimate the magnetic field distribution of the blink artifact by principal component analysis (PCA) [25]. The PCA component explaining the majority of the variance in the averaged magnetic field epoch corresponding to the blink artifact was saved for later use. The length of the epoch for the evoked response analysis was 400 ms before and 2000 ms after the stimulus onset. The 200 ms segment prior to stimulus onset was set as baseline. Epochs were low-pass filtered at 20 Hz (zero-phase shift Butterworth filter, 24 dB/oct) for further analysis.

### Analysis

The source analysis was done using BESA software (BESA Research 5.3.7, BESA GmbH, Germany). For 3D reconstruction of the brain surface for each subject, we used individual's magnetic resonance imaging (Siemens Allegra, 3.0-T). A head position indicator system was used for the MEG-MRI alignment. Dipole locations were transformed into Talairach coordinates by BESA and Brain Voyager (QX 1.4, Maastricht, The Netherlands) softwares. For each subject, SF sources were estimated by fitting two unconstrained dipoles, one in each hemisphere, to the epoch beginning at 600 ms and ending at 950 ms after the stimulus onset. To increase the signal to noise ratio of the evoked responses, the average of all the conditions were used for dipole fitting. The dipole model was then used as a spatial filter for generating the SF source waveforms for each condition [26]. In order to compensate for the ocular artifacts during source wave generation, the PCA component that represents the magnetic field distribution of the blink artifacts was also modeled to eliminate the ocular artifacts in addition to the two-dipole model. SF amplitudes were calculated as mean amplitude values over the interval of 600 - 950 ms after stimulus onset for each source and each condition. We analyzed the SF amplitudes using repeated measures analysis of variance (ANOVAs). First, in order to compare the SF amplitudes evoked by RFNs to that of white noise, we performed ANOVAs separately for each hemisphere. Then, to account for subject and hemispheric variability in SF amplitudes, we normalized the values obtained from each hemisphere with respect to the means of amplitude values in the WN, 5-, 20-, 50-, 200-, and 500-RFN conditions. We analyzed the rate effect using the normalized amplitudes from 24 hemispheres. For this analysis, we used only the WN, 5-50-, and 500 Hz conditions. As the Mauchley sphericity tests that were run prior to each ANOVA showed that the assumption of sphericity was valid, no correction for the degrees of freedom was necessary. The effect size was given as partial eta squared $\left({ŋ}_{p}^{2}\right)$, which reflects the sum of squares of the effect analyzed divided by the total of the effect and error sum of squares. For post hoc comparisons among the conditions, Bonferroni corrected t-tests were performed. Planned comparisons of 10-and 200 Hz conditions were done by uncorrected paired t-test. An alpha level of 0.05 was adopted for significance.

## Results

**Figure 2:**
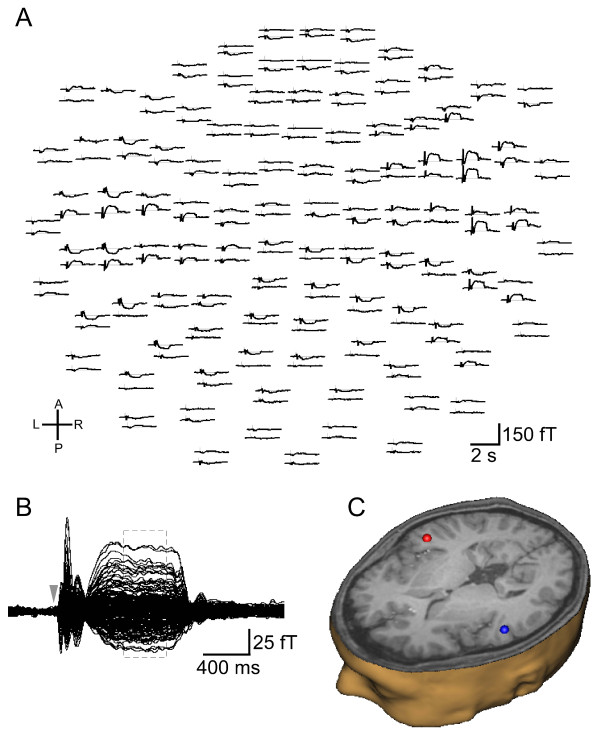
**Sustained field response in a representative subject**. A) Top view of sensor waveforms showing the average of all trials in a representative subject (A, anterior; P, posterior; L, left; R, right). B) Sensor data seen in A are superimposed. Dashed lines indicate dipole fitting interval (600 - 950 ms). Gray arrowhead shows the stimulus onset. C) Estimated dipole locations for the sustained field (SF) response overlaid on the representative subject's MRI.

Representative data in Figure 2 show the sensor waveforms averaged across all the conditions and the dipole localizations for SF. SF amplitudes were calculated as the mean amplitude over the epoch 600 - 950 ms (dotted lines in Figure 2B) after the stimulus onset.

**Figure 3:**
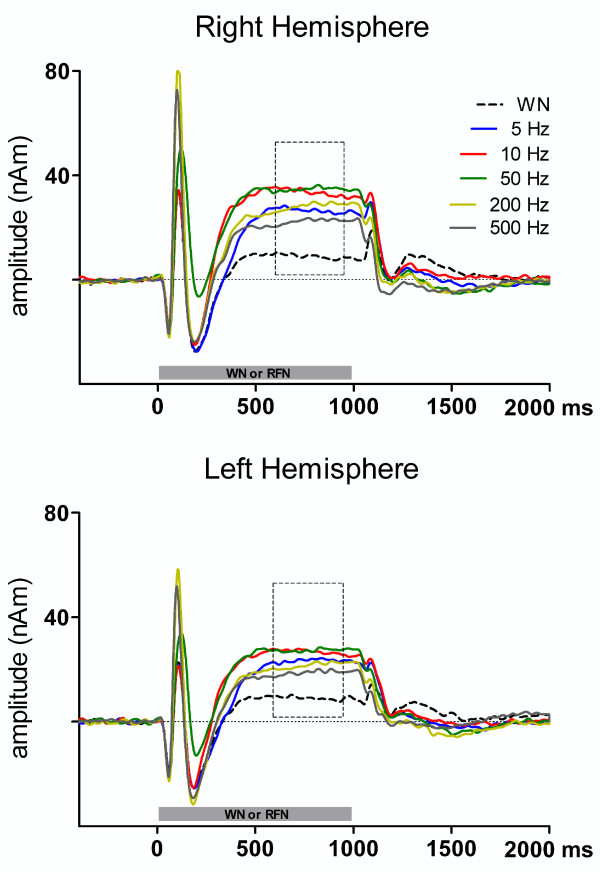
**Grand averaged sourcewaveforms**. Grand averaged sourcewaveforms for each stimulus condition. Dashed rectangles (600 - 950 ms) show the interval for mean sustained field (SF) amplitude measurement (n = 12). Upper and lower graphs indicate the SF source strengths in right and left hemispheres. Horizontal gray rectangles represent sound stimuli. (WN, white noise, RFN, repeated frozen noises).

The two-dipole model produced stable dipoles that were located close to the lateral border of the primary auditory cortex within the 10 - 20% probability region [27]. The average Talairach coordinates and their standard deviations (SD) were × = 49 (SD = 4), y = - 14 (SD = 4), z = 7 (SD = 5) for the right hemisphere and × = 49 (SD = 5), y = -20 (SD = 3), z = 6 (SD = 4) for the left hemisphere. The grand average source waveforms for each condition are shown in Figure 3.

One-factor ANOVA analysis of normalized source strengths of the sustained fields resulted in significant F values for both hemispheres (right hemisphere, F(5,55) = 55.57, p < 0.001, ${ŋ}_{p}^{2}=0.84$; left hemisphere, F(5,55) = 38.61, p < 0.001, ${ŋ}_{p}^{2}=0.78$). Bonferroni corrected t-tests revealed that SF amplitudes for all RFNs were significantly larger than for white noise for both hemispheres (right hemisphere, for 5 Hz, t = 9.47, p < 0.001, for 10 Hz, t = 11.44, p < 0.001, for 50 Hz, t = 13.10, p < 0.001, for 200 Hz, t = 10.58, p < 0.001, for 500 Hz, t = 6.87, p < 0.001, left hemisphere, for 5 Hz, t = 9.00, p < 0.001, for 10 Hz, t = 11.05, p < 0.001, for 50 Hz, t = 11.79, p < 0.001, for 200 Hz, t = 7.51, p < 0.001, for 500 Hz, t = 6.90, p < 0.001).

**Figure 4:**
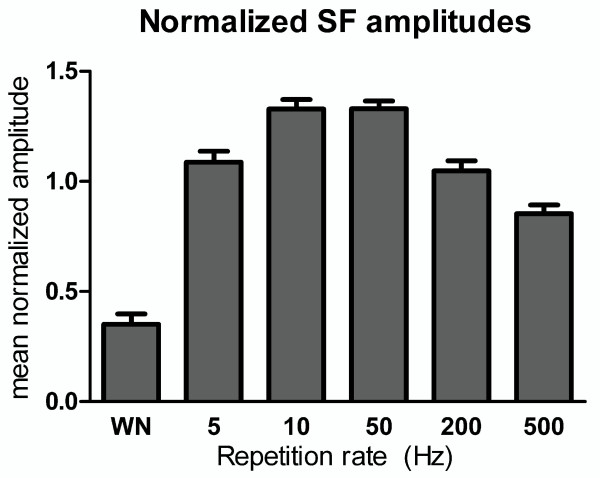
**Mean normalized source strengths of the sustained fields (SF)**. Mean normalized source strengths of the sustained fields (SF) for the interval 600 - 950 ms post stimulus onset (mean ± standard error, n = 24 (hemispheres)). The x-axis represents the repetition rates of the noises (WN (white noise), 5-, 10-, 50-, 200-, and 500 Hz repeated frozen noises (RFNs)).

Figure 4 shows the mean values of normalized SF amplitudes that were averaged across 24 hemispheres. Error bars indicate the standard error of mean and the x-axis represents the stimulus conditions. One-factor ANOVA using the WN, 5-, 50- and 500 Hz conditions revealed a significant main effect of rate (F(3,69) = 116.54, p < 0.001, ${ŋ}_{p}^{2}=0.84$). Bonferroni corrected t-tests revealed that SF amplitudes showed an increase as rate was increased from 5 Hz to 50 Hz (t = 3.84, p = 0.005) and a decrease as rate was further increased to 500 Hz (t = -11.36, p < 0.001) and the responses to 5 Hz repetition rate was significantly larger than 500 Hz (t = 4.12, p = 0.003).

When the normalized SF amplitudes evoked by 10- and 200 Hz RFNs were compared using an uncorrected t-test, 10 Hz RFNs was found to evoke significantly larger SF response than 200 Hz RFNs (t = 4.47, p < 0.001).

## Discussion

In this study, we evaluated the effect of repetition rate on auditory sustained field responses using periodic and aperiodic noise stimuli. All the SF amplitudes to RFNs were significantly larger than that of WN, indicating that sensitivity to periodicity was maintained within the repetition range of 5 to 500 Hz. When we compared the 200 Hz RFN condition that has tonal quality and evokes strong pitch sensation with the 10 Hz condition, which is not perceived as a continuous tone but rather as individual repetitive events, the evoked SF responses were significantly larger for 10 Hz condition. This finding is contradicting with the previous report by Gutschalk et al. [8] where the periodicity sensitivity of SF generators were shown to be stronger for the pitch range repetition rates. The monotonic decrease of SF amplitudes as the repetition rate of transient stimuli decrease below the pitch range found by Gutschalk et al. [8] appears to be, at least partially, caused by the decrease of overall sound energy. Increased response amplitudes to 10 Hz compared to 200 Hz and to 5 Hz compared to 500 Hz also show that cortical sensitivity to low frequency fluctuations below the pitch range is not specific to vowel stimulation [16] but persists because of periodicity detection. Using Dutch syllables and meaningful sentences, Drullman et al. [13,28] showed that temporal modulation frequencies between 4 and 16 Hz were essential for speech intelligibility since the understanding of sentences was not influenced when the temporal envelope of speech is high-pass filtered at 4 Hz or low-pass filtered at 16 Hz. They also reported that reducing slow modulations affected identification of consonants more than vowels [13,28]. Arai et al. [29] extended Drullman's experiments by using additional band-pass filtering and showed that when components of modulation spectrum between 1 and 16 Hz were preserved, the intelligibility of Japanese syllables was not severely impaired. The persistence of periodicity sensitivity for low frequency repetition rates suggest that in addition to high frequency periodicity that provides clues for voice pitch, regularity-sensitive SF generators can effectively detect slow modulations that are important for speech perception.

We observed significant differences between the response amplitudes within the repetition frequency range. First, SF amplitudes increased when repetition rate was increased from 5 Hz to 50 Hz, then the amplitudes decreased as rate was increased further to 500 Hz. This complex response pattern might be due to various factors. Perception of the low frequency RFNs (< 20 Hz) depends on identification of the repetitive temporal patterns since the harmonics of the sound cannot be resolved in the periphery [24]. As the repetition frequency increases, RFNs start to carry strong spectral cues in addition to temporal cues, as their fundamental and/or lower harmonics are resolved. Enhanced responses to 50 Hz repetition rate might represent simultaneous temporal and spectral processing in the auditory cortex [15]. The increase in response amplitudes as the rate was increased from 5 Hz to 50 Hz may also be the effect of the number of repeating segments. For constant stimulus length, which was one second in our study, only the four repetitive segments in 5 Hz condition would be perceived as periodic. The difference between the number of repeating segments that evoke periodicity perception would have caused the variation of the response amplitudes. The stronger responses for 50 Hz compared to 500 Hz condition could be related to an increased neural activity in the auditory cortex as a result of increased spectral complexity within the spectral envelope [30,31]. Higher spectral density of the 50 Hz RFNs due to closely spaced harmonics could have resulted in stronger SF responses.

The lowest repetition rate reported in previous electrophysiological studies was 9 Hz vowel stimulation [16], which showed 9 Hz transient response peaks overlaid on the steady SF. Multiple transients would correspond to the temporal changes in amplitude envelope, i.e. energy onset at the transition of silence to sound within the 9 Hz vowel sequence. As the RFN stimuli contain no silent periods between the repeating segments and no periodic amplitude envelope, we did not observe any energy-onset transient responses. However, there can be another explanation for the lack of transient responses in our data. It has been shown that when repeating noise segments are separated with random noise of shorter length, N1-like deflections, which are time-locked to the onsets of the repeating segments, are elicited [32]. Although repetitive frozen noises do not have any clues regarding the onset of the segments, psychophysical studies showed that the spectro-temporal basis of the perceived rhythmic acoustic events in continuous RFNs is restricted to about 100 ms segments [20,21]. If the transient responses that are superimposed on SF represent phase-specific responses to such events, our acquisition method and averaging could have caused the transients to smoothen out. As different RFN stimuli were used for each trial, the transient responses evoked by detection of the acoustical events would be at unpredictable points of the repeating segments and would not survive averaging.

The periodicity and sound level sensitive SF components were first described by a four-dipole model [8]. As we used a two-dipole model in this study, the modeled responses were likely to include both SF components. However, as the stimuli had equal energy, the contribution of the energy-sensitive component would also be the same among the conditions. Therefore, the obtained results mainly reflected the periodicity-sensitive SF component.

## Conclusions

The analysis of SF amplitudes evoked by periodic noises revealed that periodicity sensitivity of SF generators is maintained below the pitch range and exists even in case of artificial basic sounds. The persistence of sensitivity to periodic sound stimuli with repetition rates as low as 5 Hz suggests that SF generators may play a role in processing the slow amplitude modulations that are important for speech perception.

## Authors' contributions

SK contributed to planning the study, data collection, analysis, and drafting the paper. KI and HO contributed to planning the study and drafting the paper. NO contributed to data collection and analysis. RK contributed to drafting the paper. All authors read and approved the final manuscript.

## Supplementary Material

Additional file 1 **Frequency spectrum and samples of the sound stimuli**. One sample from each stimulus set is embedded (marked with loudspeaker icons) in the corresponding frequency spectrum representation. For each condition, the frequency spectrum is calculated over all the stimuli in the set. Sound samples were recorded from the earpiece of the sound delivery system. (WN, white noise). Click here for file
