# Supplementary figures and images for: Auditory sustained field responses to periodic noise
Source: BMC Neurosci. 2012 Jan 6;13:7. doi: 10.1186/1471-2202-13-7 (PMC3293709; doi:10.1186/1471-2202-13-7)

## Supplementary Figure

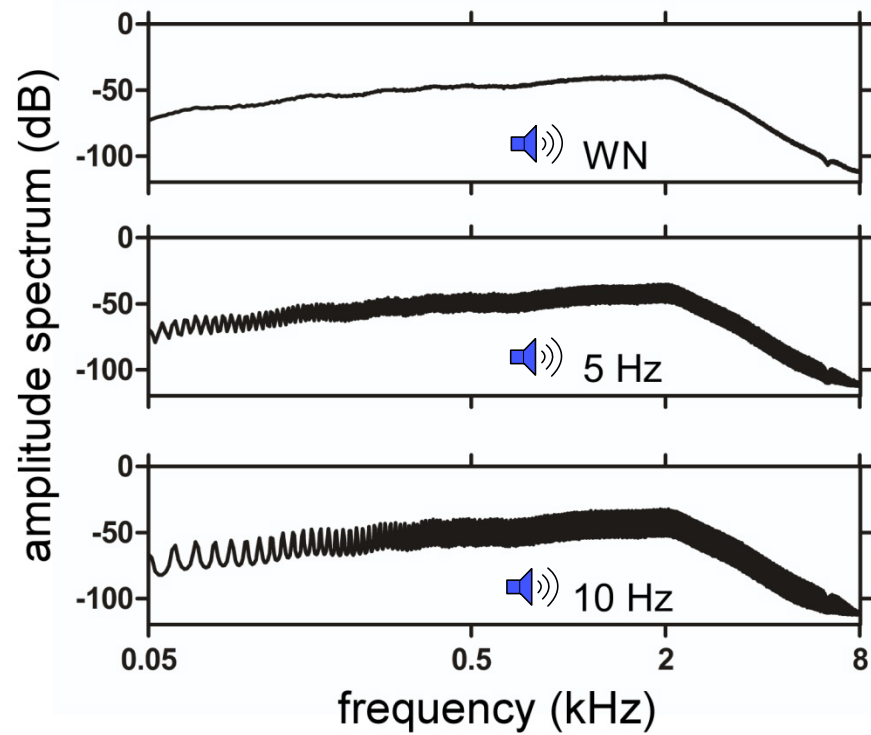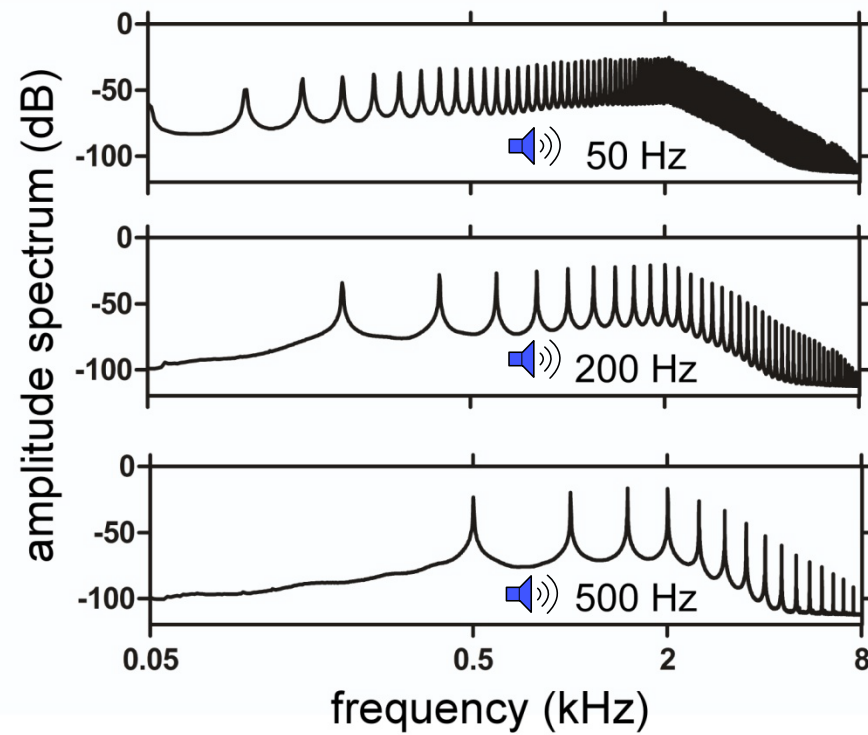

Supplement: Additional file 1 — Frequency spectrum and samples of the sound stimuli. One sample from each stimulus set is embedded (marked with loudspeaker icons) in the corresponding frequency spectrum representation. For each condition, the frequency spectrum is calculated over all the stimuli in the set. Sound samples were recorded from the earpiece of the sound delivery system. (WN, white noise). [file 1471-2202-13-7-S1.PDF]
